# Supplementary material for: Learning curve and surgical outcome of robotic assisted colorectal surgery with ERAS program
Source: Sci Rep. 2022 Nov 29;12:20566. doi: 10.1038/s41598-022-24665-w (PMC9709162; doi:10.1038/s41598-022-24665-w)
Supplement: Supplementary file 1 — Supplementary Information 1. [file 41598_2022_24665_MOESM1_ESM.zip › LSC Raw data-Ñ[▒K/122/H_ñw╝╨░O▒Kñσ.pdf]

正式摘要(電子簽章：000000212771759)

W52 / W52 ☐ 英文標題(注意:無英文姓名-請補病人基本檔!) ☐【讀取】憑證

臺中榮民總醫院

出院病歷摘要

轉入號： 轉出號：

[redacted]

(8)流水編號	
(9)入院日期	110/03/17 (2021) 直腸外科 W52 - 102
(10)轉科日期	
(11)出院日期	110/03/22 (2021) 住院天數計 5 日

診斷

- 入院：1. Colon cancer, at hepatic flexture, pending pathology
  [Hx]
  1. HTN
  2. Hyperlipidemia
  3. TIA under bokey
- 出院：1. Colon cancer, at hepatic flexture, pending pathology, s/p Lap RH with anatomosis on 2021/03/18

主訴

- Colon cancer noted at LMD

病史

- This 65 y/o female with a hx of
  1. HTN
  2. Hyperlipidemia
  3. TIA under bokey
  This time, stool OB was noted during health examination in 2020/10. Colonscopy in 2020/11 showed 1. one polypoid tumor, with bleeding and luminal narrowing, at about 80cm proximal to AV. (maybe hepatic flexture)---biopsy (A) 2. small polyps at about 10cm (x2#) and 20cm (x4#) proximal to AV. 3. mixed hemorrhoids with erosions. Abd CT was also arranged and showed colon cancer at hepatic flexture and no regional lymph node metastasis was reported. Under the impression of colon cancer, she was admitted for LAP RH with ERAS protocol.

體檢發現

- Anal inspection:
  Recto digital examination:
  Sigmoidoscopy:
  Anoscopy:
  Abdomen:
  - General appearance :
    - easy looking , conscious clear , without cardiopulmonary distress
    - Vital signs自動帶入date 體溫(BT) 脈搏(P) 呼吸(R) 血壓(BP) 身高(BH) 體重(BW) BMI
      20210317 36℃ 107次/分 19次/分 151/78mmHg 145.5cm 45.7kg 21.6
  - HEENT :
    - Eyes: no anemic conjunctiva, no icteric sclera, normal EOM
    - ENT: no otorrhea, no rhinorrhea, no erythematous mucosa, no injected tonsil, no deformity
  - Neck: supple, freely movable, trachea midline,no deformity, no engorged jugular vein , no palpable thyroid or lymph node enlargement
  - Chest:
    - Inspection: Grossly normal, symmetric and fully expansion, no accessory muscle used, no operation scar, no spider angiomata, no gynecomastia
    - Palpation: no palpable mass or nodule, no subcutaneous emphysema, no

- tenderness
  - Ausculation: clear breathing sound
- Heart:
  - Percussion: normal heart size
  - Ausculation: regular heart beat, normal S1, S2, no gallop, no murmur
- Abdomen:
  - Inspection: in
    - flat contour, symetrically, no scar, no stria, no dilated veins, no rashes
  - Ausculation: normoactive bowel sound, no abdominal bruits
  - palpation: soft, no muscle guarding or rigidity, no local tenderness, no rebounding pain, no palpable mass, no palpable pulsation
  - Percussion: no shifting dulllness, no hepatomegaly, no splenomagaly
-

Peripheral pulsation :	CA	BA	RA	FA	PA	PTA	DPA
Right side :	++	++	++	++	++	++	++/+++
Left side :	++	++	++	++	++	++	++/+++

- Genito-anus: no tenderness or palpable mass lesion or rectal shelf, normal external genitalia, normal anal tone, brown stool

CRS CONDITION

-
  - Lung

手術日期及方法

- 手術日期及方法20210318 1. Laparoscopic right hemicolectomy with side to side anastomosis (OpenGIA 75-1.5 X2 were applied)
  2. Ileo-colic artery, right colic artery and right branch of middle colic artery were all ligated via Hemo-clips.
  3. Take down of hepatic flexure : (+)
  4. Cut margin: proximal (ileum) 6 cm, (colon) >15 cm, distal 5 cm.
  5. Repair of mesentery defect : (+). Done via continuous running suture with 3-O mersilk.
  6. No drainage tube was placed.
  7. ADHESION BARRIER FILM was placed below the central abdominal wound.

住院治療經過

- After admission, no contraindication was noted for Lap RH. Neurologist was also consulted for Aspirin use and add back whithin 1 week after the operation was suggested. The operation was then performed smoothly on 2021/03/18 with anastomosis. On 03/19, she started liquid diet and started BLAND III on 03/20. She tolerated well. The surgical wounds were clean and no discharge. Since she was under a relatively stable condition, she was discharged on 03/22. OPD f/u was also arranged.

合併症

一般檢查

- 累積報告 :

DATE	TIME	ALB	TP	BIL.T	BIL.D	ALKP	AST	ALT	LDH	CHOL	TG	HDL-C	LDL	UA	NH3	AMY
1100317	1145	4.4		0.8	0.2	193	17	16	175							
1100317	2139		6.8							158	44	60	87	3.5		
1100318	1620	3.5		0.9			22	13								
1100322	0933	3.3	5.8	0.51	0.15	140	18	10	185							

DATE	TIME	WBC	RBC	HGB	HCT	MCV	PLT	BLST	NEUT	BAND	SEG	LYM	MON	EOS	BAS
1100317	1145	8480	3.54	8.6	27.8	78.5	426		67.9			25.5	5.0	1.2	0.4
1100318	1623	13920		9.5	29.2		291		92.9			5.8	1.1	0.1	0.1
1100322	0921	8280	4.00	10.2	32.3	80.8	291		70.4			18.4	5.8	5.2	0.2

DATE	TIME	NA	K	CL	CA	BUN	CREAT	Mg	P	CRP	ESR	PCT	LIPASE	LACTATE
1100317	1145	142	3.8		9.7	14	0.83							
1100317	2139									0.113				
1100318	1620	142	3.2		8.1	9	0.66							
1100322	0933	142	3.4	103	8.2	7	0.68							

DATE	TIME	GLUCOSE	PAB	TRF	ZN	CU	KETONE	CK	CKMB	PT-P	PT-C	APTT-P	APTT-C	TRO-T
1100317	1145	94								10.6	10.6	24.9		
1100319	2205													5.86
1100319	2206							168	3					

特殊檢查

病理報告

- pending

其它

出院時情況

- 3.改門診治療

出院指示

藥名	劑量	劑量單位	用法	頻次	數量	數量單位	現況	天數	單位
(口)Takepron OD tab 30mg	1	TAB	PO	QDAC	7	TAB	TH	7	天
Acetal tab 500mg	1	TAB	PO	Q6HPRN	28	TAB	TH	7	天

出院計劃

主治醫師_2062F 蔣鋒帆_ / 住院醫師_9757K 翁明謙_

正式摘要**(**電子簽章：**000000212771759)**

W52 / W52

doctorRank:B
